# 3,5-Bis[1-acetyl-5-(4-chloro­phen­yl)-4,5-dihydro-1*H*-pyrazol-3-yl]-2,6-dimethyl­pyridine tetra­hydro­furan solvate

**DOI:** 10.1107/S1600536808018497

**Published:** 2008-06-25

**Authors:** Qun Qian, Jun Zhang, Min Zhang, Xiang He, YiBen Xia

**Affiliations:** a Department of Chemistry, College of Science, Shanghai University, Shanghai 200444, People’s Republic of China; b School of Materials Science and Engineering, Shanghai University, Shanghai 200072, People’s Republic of China

## Abstract

In the title compound, C_29_H_27_Cl_2_N_5_O_2_·C_4_H_8_O, the polycyclic system is composed of three parts: one central pyridine ring substituted by two functionalized pyrazoline rings. The dihedral angles between the central pyridine plane and pyrazoline planes are 5.11 (1) and 13.99 (1)°, whereas the dihedral angles between each chloro­phenyl plane and the attached pyrazoline planes are 88.65 (1) and 83.87 (1)°. Mol­ecules are linked by inter­molecular C—H⋯O hydrogen bonds, forming a three-dimensional network.

## Related literature

For related literature, see: Holla *et al.* (2002 ▶); Palaska *et al.* (1996 ▶); Soudi *et al.* (2005 ▶); Chopra *et al.*(2006 ▶).
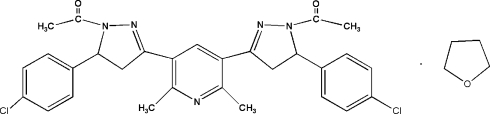


## Experimental

### 

#### Crystal data

C_29_H_27_Cl_2_N_5_O_2_·C_4_H_8_O
*M* *_r_* = 620.56
Monoclinic, 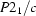

*a* = 16.888 (3) Å
*b* = 11.180 (2) Å
*c* = 17.313 (4) Å
β = 98.69 (3)°
*V* = 3231.5 (11) Å^3^
*Z* = 4
Mo *K*α radiation
μ = 0.24 mm^−1^
*T* = 293 (2) K
0.20 × 0.20 × 0.10 mm

#### Data collection

Bruker SMART CCD area-detector diffractometer
Absorption correction: multi-scan (*SADABS*; Sheldrick, 1996 ▶) *T*_min_ = 0.953, *T*_max_ = 0.976
16422 measured reflections
5715 independent reflections
2714 reflections with *I* > 2σ(*I*)
*R*_int_ = 0.037

#### Refinement

*R*[*F* ^2^ > 2σ(*F* ^2^)] = 0.055
*wR*(*F* ^2^) = 0.171
*S* = 0.95
5715 reflections
393 parameters
H-atom parameters constrained
Δρ_max_ = 0.31 e Å^−3^
Δρ_min_ = −0.36 e Å^−3^

### 

Data collection: *SMART* (Bruker, 2000 ▶); cell refinement: *SAINT* (Bruker, 2000 ▶); data reduction: *SAINT*; program(s) used to solve structure: *SHELXS97* (Sheldrick, 2008 ▶); program(s) used to refine structure: *SHELXL97* (Sheldrick, 2008 ▶); molecular graphics: *SHELXTL* (Sheldrick, 2008 ▶); software used to prepare material for publication: *SHELXTL*.

## Supplementary Material

Crystal structure: contains datablocks I, global. DOI: 10.1107/S1600536808018497/bh2175sup1.cif

Structure factors: contains datablocks I. DOI: 10.1107/S1600536808018497/bh2175Isup2.hkl

Additional supplementary materials:  crystallographic information; 3D view; checkCIF report

## Figures and Tables

**Table 1:** Hydrogen-bond geometry (Å, °)

*D*—H⋯*A*	*D*—H	H⋯*A*	*D*⋯*A*	*D*—H⋯*A*
C9—H9*B*⋯O3	0.97	2.49	3.423 (7)	163
C21—H21*B*⋯O1^i^	0.97	2.57	3.211 (4)	123
C9—H9*A*⋯O1^i^	0.97	2.35	3.234 (4)	151
C23—H23*A*⋯O2^ii^	0.96	2.45	3.385 (4)	166

Symmetry codes: (i) 
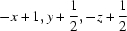
; (ii) 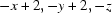.

**Fig. 1.**
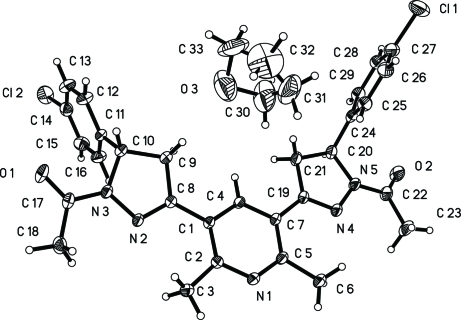
The molecular structure of the title molecule, with the atom-numbering scheme. Displacement ellipsoids are drawn at the 15% probability level.

**Fig. 2.**
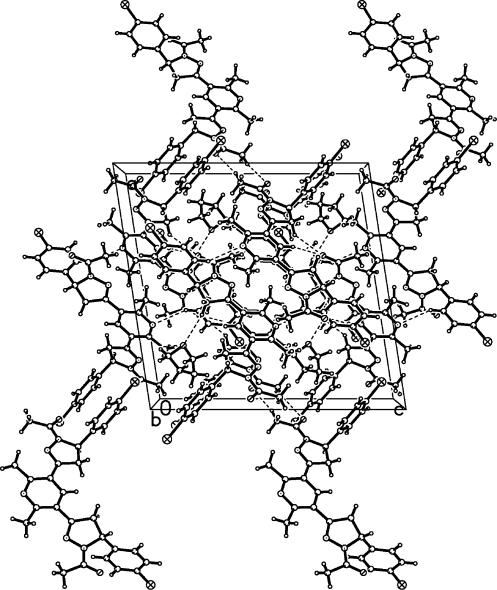
Crystal packing diagram of compound (I). Hydrogen bonds are indicated by dashed lines.

## supplementary crystallographic information

### Comment

Nitrogen-containing heterocycles compounds are well known natural products moieties which present interesting biological activities and pharmacological properties (Holla *et al.*, 2002; Soudi *et al.*, 2005). For example, 1,3,5-trisubstitituted pyrazolines show reversible and selective monoamine oxidase inhibitory properties. Their selective biological activity is in part due to the influence of substitution on the compounds conformation (Palaska *et al.*, 1996). These useful applications for the 1,3,5-trisubstituted pyrazolines attracted our attention and we present here a new member of this family.

The molecular structure of (I) consists of one polycyclic molecule and one tetrahydrofuran solvent molecule (Fig. 1). There are two substituted phenyl rings bonded with two different pyrazoline rings, and these two pyrazoline rings are further bonded with one central pyridine ring. The dihedral angles between the pyridine plane and the two pyrazoline planes are 5.10 and 13.99°. Each substituted phenyl plane is nearly normal to the corresponding pyrazoline plane, with dihedral angles of 88.04 and 83.38°. Bond lengths in the pyrazoline rings and substituted phenyl rings are in good agreement with those found in similar compounds (*e.g*. Chopra *et al.*, 2006).

In the crystal structure, there are three types of intermolecular and one intramolecular hydrogen bonds, which make the crystal structure to be more stable (see hydrogen-bond geometry Table).

### Experimental

2,6-Dimethyl-3,5-di-[3-(4-chlorophenyl)-acryloyl-pyridine (1 mmol, 0.436 g), and 85% hydrazine hydrate solution (4 mmol, 0.235 g) were dissolved in 5 mL of acetic acid. The mixture was refluxed for 8 h, and then allowed to cool to room temperature. The reaction mixture was poured into crushed ice, and neutralized with diluted NaOH solution. The solid separated was filtered off, washed with water, dried and recrystallized from ethyl acetate, to give a colourless compound in a yield of of 42% (m.p. 489–491 K). Single crystals suitable for X-ray analysis were obtained form tetrahydrofuran at room temperature.

### Refinement

All H atoms were placed in calculated positions, with C—H = 0.93–0.98 Å, and included in the final cycles of refinement using a riding model and *U*_iso_(H) = 1.2–1.5*U*_eq_(C). The THF solvate molecule has high displacement parameters, suggesting that the molecule is probably disordered over a number of positions.

### Crystal data

**Table:**

C_29_H_27_Cl_2_N_5_O_2_·C_4_H_8_O	*F*_000_ = 1304
*M**_r_* = 620.56	*D*_x_ = 1.276 Mg m^−^^3^
Monoclinic, *P*2_1_/*c*	Melting point = 489–491 K
Hall symbol: -P 2ybc	Mo *K*α radiation λ = 0.71073 Å
*a* = 16.888 (3) Å	θ = 2.2–20.8º
*b* = 11.180 (2) Å	µ = 0.24 mm^−^^1^
*c* = 17.313 (4) Å	*T* = 293 (2) K
β = 98.69 (3)º	Prism, colourless
*V* = 3231.5 (11) Å^3^	0.20 × 0.20 × 0.10 mm
*Z* = 4	

### Data collection

**Table:**

Bruker SMART CCD area-detector diffractometer	5715 independent reflections
Radiation source: fine-focus sealed tube	2714 reflections with *I* > 2σ(*I*)
Monochromator: graphite	*R*_int_ = 0.037
*T* = 293(2) K	θ_max_ = 25.1º
ω scans	θ_min_ = 2.2º
Absorption correction: multi-scan(SADABS; Sheldrick, 1996)	*h* = −20→17
*T*_min_ = 0.953, *T*_max_ = 0.976	*k* = −13→13
16422 measured reflections	*l* = −14→20

### Refinement

**Table:**

Refinement on *F*^2^	Hydrogen site location: inferred from neighbouring sites
Least-squares matrix: full	H-atom parameters constrained
*R*[*F*^2^ > 2σ(*F*^2^)] = 0.055	*w* = 1/[σ^2^(*F*_o_^2^) + (0.0811*P*)^2^] where *P* = (*F*_o_^2^ + 2*F*_c_^2^)/3
*wR*(*F*^2^) = 0.171	(Δ/σ)_max_ < 0.001
*S* = 0.95	Δρ_max_ = 0.31 e Å^−^^3^
5715 reflections	Δρ_min_ = −0.36 e Å^−^^3^
393 parameters	Extinction correction: SHELXL97 (Sheldrick, 2008), Fc^*^=kFc[1+0.001xFc^2^λ^3^/sin(2θ)]^-1/4^
Primary atom site location: structure-invariant direct methods	Extinction coefficient: 0.0020 (6)
Secondary atom site location: difference Fourier map	

### Fractional atomic coordinates and isotropic or equivalent isotropic displacement parameters (Å^2^)

**Table:**

	*x*	*y*	*z*	*U*_iso_*/*U*_eq_	
C1	0.60818 (16)	0.4491 (2)	0.07953 (16)	0.0487 (7)	
C2	0.61080 (17)	0.4038 (2)	0.00456 (17)	0.0545 (8)	
C3	0.5585 (2)	0.3053 (3)	−0.03305 (18)	0.0768 (10)	
H3A	0.5763	0.2822	−0.0810	0.115*	
H3B	0.5041	0.3327	−0.0438	0.115*	
H3C	0.5617	0.2378	0.0015	0.115*	
N1	0.66326 (15)	0.4459 (2)	−0.03987 (13)	0.0595 (7)	
C5	0.71460 (17)	0.5337 (3)	−0.01436 (16)	0.0567 (8)	
C6	0.7692 (2)	0.5687 (3)	−0.07116 (18)	0.0774 (10)	
H6A	0.7591	0.5185	−0.1165	0.116*	
H6B	0.8239	0.5594	−0.0470	0.116*	
H6C	0.7597	0.6507	−0.0863	0.116*	
C7	0.71516 (16)	0.5856 (2)	0.05992 (16)	0.0497 (7)	
C8	0.55499 (17)	0.4029 (3)	0.13232 (16)	0.0515 (7)	
C9	0.56623 (19)	0.4295 (3)	0.21808 (17)	0.0738 (10)	
H9A	0.5641	0.5149	0.2275	0.089*	
H9B	0.6170	0.3986	0.2440	0.089*	
C10	0.49590 (18)	0.3651 (3)	0.24637 (16)	0.0605 (8)	
H10	0.5166	0.3034	0.2843	0.073*	
C11	0.43991 (17)	0.4448 (3)	0.28162 (16)	0.0556 (8)	
C12	0.4319 (2)	0.4375 (3)	0.35906 (19)	0.0790 (10)	
H12	0.4622	0.3818	0.3906	0.095*	
C13	0.3796 (2)	0.5111 (3)	0.3915 (2)	0.0890 (11)	
H13	0.3751	0.5048	0.4442	0.107*	
C14	0.3353 (2)	0.5921 (3)	0.3460 (2)	0.0743 (10)	
C15	0.3422 (2)	0.6030 (3)	0.2690 (2)	0.0839 (11)	
H15	0.3123	0.6597	0.2380	0.101*	
C16	0.3946 (2)	0.5282 (3)	0.23771 (19)	0.0773 (10)	
H16	0.3989	0.5352	0.1850	0.093*	
C17	0.40085 (19)	0.2213 (3)	0.1693 (2)	0.0646 (9)	
C18	0.3759 (2)	0.1615 (3)	0.0924 (2)	0.0840 (11)	
H18A	0.4167	0.1062	0.0826	0.126*	
H18B	0.3683	0.2206	0.0517	0.126*	
H18C	0.3266	0.1191	0.0934	0.126*	
C19	0.76779 (17)	0.6835 (2)	0.09060 (17)	0.0533 (7)	
C20	0.82785 (18)	0.8467 (3)	0.16815 (18)	0.0634 (8)	
H20	0.7995	0.9234	0.1628	0.076*	
C21	0.7666 (2)	0.7438 (3)	0.16844 (18)	0.0712 (9)	
H21A	0.7830	0.6890	0.2113	0.085*	
H21B	0.7137	0.7746	0.1726	0.085*	
C22	0.9101 (2)	0.8921 (3)	0.0629 (2)	0.0842 (11)	
C23	0.9350 (3)	0.8490 (4)	−0.0120 (2)	0.1197 (17)	
H23A	0.9754	0.9012	−0.0265	0.179*	
H23B	0.8894	0.8489	−0.0526	0.179*	
H23C	0.9560	0.7693	−0.0049	0.179*	
C24	0.89355 (19)	0.8516 (3)	0.23768 (18)	0.0601 (8)	
C25	0.9417 (2)	0.7542 (3)	0.2596 (2)	0.0760 (10)	
H25	0.9321	0.6827	0.2324	0.091*	
C26	1.0037 (3)	0.7608 (4)	0.3210 (2)	0.0918 (12)	
H26	1.0359	0.6945	0.3353	0.110*	
C27	1.0171 (3)	0.8655 (5)	0.3602 (2)	0.0953 (12)	
C28	0.9697 (3)	0.9627 (4)	0.3417 (2)	0.0921 (12)	
H28	0.9789	1.0331	0.3703	0.111*	
C29	0.9079 (2)	0.9559 (3)	0.2799 (2)	0.0767 (10)	
H29	0.8755	1.0223	0.2667	0.092*	
C30	0.7975 (7)	0.4446 (8)	0.3062 (6)	0.264 (6)	
H30A	0.7778	0.4645	0.2521	0.317*	
H30B	0.8069	0.5180	0.3359	0.317*	
C31	0.8602 (6)	0.3835 (13)	0.3112 (7)	0.272 (6)	
H31A	0.9071	0.4317	0.3291	0.327*	
H31B	0.8659	0.3474	0.2614	0.327*	
C32	0.8479 (8)	0.2933 (8)	0.3685 (10)	0.309 (7)	
H32A	0.8321	0.2176	0.3435	0.371*	
H32B	0.8961	0.2815	0.4060	0.371*	
C33	0.7871 (7)	0.3404 (12)	0.4042 (4)	0.259 (5)	
H33A	0.7612	0.2811	0.4327	0.311*	
H33B	0.8041	0.4083	0.4374	0.311*	
Cl1	1.09785 (9)	0.87718 (15)	0.43586 (8)	0.1632 (7)	
Cl2	0.26751 (7)	0.68157 (10)	0.38565 (7)	0.1173 (5)	
C4	0.66139 (16)	0.5401 (2)	0.10563 (15)	0.0507 (7)	
H4A	0.6610	0.5716	0.1553	0.061*	
N2	0.49515 (14)	0.3347 (2)	0.10922 (13)	0.0552 (6)	
N3	0.45775 (15)	0.3068 (2)	0.17305 (14)	0.0623 (7)	
N4	0.82032 (15)	0.7275 (2)	0.05271 (14)	0.0632 (7)	
N5	0.85993 (15)	0.8201 (2)	0.09521 (14)	0.0677 (7)	
O1	0.37297 (14)	0.19533 (19)	0.22848 (14)	0.0840 (7)	
O2	0.93475 (17)	0.9838 (2)	0.09668 (14)	0.1037 (9)	
O3	0.7430 (3)	0.3717 (6)	0.3371 (5)	0.261 (3)	

### Atomic displacement parameters (Å^2^)

**Table:**

	*U*^11^	*U*^22^	*U*^33^	*U*^12^	*U*^13^	*U*^23^
C1	0.0502 (18)	0.0502 (17)	0.0481 (17)	−0.0035 (14)	0.0149 (13)	0.0042 (13)
C2	0.0529 (19)	0.0607 (19)	0.0503 (18)	−0.0040 (15)	0.0093 (15)	0.0033 (14)
C3	0.086 (3)	0.090 (2)	0.057 (2)	−0.029 (2)	0.0194 (17)	−0.0155 (17)
N1	0.0638 (17)	0.0687 (17)	0.0483 (15)	−0.0095 (14)	0.0156 (13)	−0.0002 (12)
C5	0.059 (2)	0.065 (2)	0.0502 (19)	−0.0019 (17)	0.0198 (15)	0.0054 (15)
C6	0.079 (2)	0.097 (3)	0.062 (2)	−0.018 (2)	0.0332 (18)	−0.0049 (18)
C7	0.0493 (18)	0.0514 (17)	0.0509 (18)	−0.0021 (14)	0.0160 (14)	0.0049 (13)
C8	0.0512 (19)	0.0568 (18)	0.0483 (18)	−0.0031 (15)	0.0130 (14)	0.0025 (14)
C9	0.069 (2)	0.106 (3)	0.0494 (19)	−0.0218 (19)	0.0191 (16)	−0.0043 (17)
C10	0.063 (2)	0.071 (2)	0.0493 (18)	−0.0085 (17)	0.0149 (15)	0.0097 (15)
C11	0.060 (2)	0.065 (2)	0.0429 (18)	−0.0101 (16)	0.0134 (15)	0.0078 (15)
C12	0.101 (3)	0.085 (3)	0.053 (2)	0.013 (2)	0.0188 (19)	0.0137 (18)
C13	0.120 (3)	0.094 (3)	0.059 (2)	0.003 (3)	0.032 (2)	0.003 (2)
C14	0.074 (2)	0.069 (2)	0.082 (3)	−0.0083 (19)	0.017 (2)	−0.0141 (19)
C15	0.091 (3)	0.076 (2)	0.080 (3)	0.008 (2)	−0.001 (2)	0.005 (2)
C16	0.088 (3)	0.090 (3)	0.053 (2)	0.006 (2)	0.0098 (18)	0.0104 (19)
C17	0.064 (2)	0.056 (2)	0.080 (3)	−0.0052 (17)	0.0312 (18)	0.0061 (17)
C18	0.086 (3)	0.071 (2)	0.100 (3)	−0.025 (2)	0.028 (2)	−0.013 (2)
C19	0.0510 (18)	0.0547 (18)	0.0569 (19)	−0.0065 (15)	0.0166 (15)	0.0053 (14)
C20	0.061 (2)	0.059 (2)	0.074 (2)	−0.0064 (16)	0.0248 (17)	−0.0054 (16)
C21	0.067 (2)	0.079 (2)	0.074 (2)	−0.0209 (18)	0.0321 (17)	−0.0136 (17)
C22	0.088 (3)	0.090 (3)	0.078 (3)	−0.042 (2)	0.026 (2)	0.006 (2)
C23	0.138 (4)	0.149 (4)	0.086 (3)	−0.075 (3)	0.063 (3)	−0.016 (3)
C24	0.065 (2)	0.058 (2)	0.062 (2)	−0.0119 (17)	0.0270 (16)	−0.0035 (16)
C25	0.087 (3)	0.065 (2)	0.078 (2)	−0.009 (2)	0.018 (2)	−0.0066 (18)
C26	0.093 (3)	0.091 (3)	0.089 (3)	0.006 (2)	0.010 (2)	0.014 (2)
C27	0.103 (3)	0.111 (3)	0.070 (3)	−0.026 (3)	0.008 (2)	0.000 (2)
C28	0.112 (3)	0.087 (3)	0.078 (3)	−0.031 (3)	0.016 (2)	−0.027 (2)
C29	0.089 (3)	0.063 (2)	0.082 (3)	−0.0087 (19)	0.026 (2)	−0.0139 (18)
C30	0.200 (9)	0.245 (10)	0.344 (12)	−0.037 (9)	0.030 (9)	0.194 (9)
C31	0.172 (8)	0.43 (2)	0.246 (11)	0.069 (10)	0.118 (8)	0.043 (10)
C32	0.337 (17)	0.183 (8)	0.391 (19)	0.106 (9)	0.002 (13)	0.113 (10)
C33	0.288 (13)	0.412 (17)	0.082 (5)	0.054 (11)	0.046 (6)	0.043 (7)
Cl1	0.1458 (12)	0.2111 (15)	0.1152 (10)	−0.0404 (11)	−0.0362 (9)	0.0005 (9)
Cl2	0.1092 (9)	0.1077 (9)	0.1404 (10)	0.0055 (7)	0.0365 (7)	−0.0408 (7)
C4	0.0535 (18)	0.0540 (18)	0.0476 (17)	−0.0036 (15)	0.0173 (14)	−0.0003 (13)
N2	0.0572 (16)	0.0591 (15)	0.0530 (15)	−0.0118 (13)	0.0199 (12)	0.0011 (12)
N3	0.0685 (18)	0.0679 (17)	0.0552 (16)	−0.0182 (14)	0.0247 (13)	−0.0002 (12)
N4	0.0627 (18)	0.0715 (17)	0.0588 (16)	−0.0186 (14)	0.0205 (13)	0.0003 (13)
N5	0.0727 (18)	0.0739 (18)	0.0610 (17)	−0.0265 (15)	0.0251 (14)	−0.0021 (14)
O1	0.0943 (18)	0.0717 (15)	0.0984 (18)	−0.0104 (13)	0.0544 (14)	0.0121 (13)
O2	0.127 (2)	0.0940 (19)	0.0980 (19)	−0.0551 (17)	0.0416 (16)	−0.0050 (15)
O3	0.146 (4)	0.291 (7)	0.330 (9)	−0.017 (4)	−0.017 (5)	0.093 (6)

### Geometric parameters (Å, °)

**Table:**

C1—C4	1.387 (3)	C19—N4	1.278 (3)
C1—C2	1.400 (4)	C19—C21	1.509 (4)
C1—C8	1.469 (4)	C20—N5	1.478 (4)
C2—N1	1.343 (3)	C20—C24	1.510 (4)
C2—C3	1.497 (4)	C20—C21	1.547 (4)
C3—H3A	0.9600	C20—H20	0.9800
C3—H3B	0.9600	C21—H21A	0.9700
C3—H3C	0.9600	C21—H21B	0.9700
N1—C5	1.340 (3)	C22—O2	1.222 (4)
C5—C7	1.410 (4)	C22—N5	1.350 (4)
C5—C6	1.498 (4)	C22—C23	1.502 (5)
C6—H6A	0.9600	C23—H23A	0.9600
C6—H6B	0.9600	C23—H23B	0.9600
C6—H6C	0.9600	C23—H23C	0.9600
C7—C4	1.388 (4)	C24—C25	1.377 (4)
C7—C19	1.459 (4)	C24—C29	1.378 (4)
C8—N2	1.281 (3)	C25—C26	1.378 (5)
C8—C9	1.498 (4)	C25—H25	0.9300
C9—C10	1.531 (4)	C26—C27	1.355 (5)
C9—H9A	0.9700	C26—H26	0.9300
C9—H9B	0.9700	C27—C28	1.359 (5)
C10—N3	1.485 (4)	C27—Cl1	1.747 (4)
C10—C11	1.495 (4)	C28—C29	1.378 (5)
C10—H10	0.9800	C28—H28	0.9300
C11—C16	1.363 (4)	C29—H29	0.9300
C11—C12	1.370 (4)	C30—C31	1.252 (10)
C12—C13	1.386 (5)	C30—O3	1.396 (8)
C12—H12	0.9300	C30—H30A	0.9700
C13—C14	1.349 (5)	C30—H30B	0.9700
C13—H13	0.9300	C31—C32	1.452 (12)
C14—C15	1.361 (5)	C31—H31A	0.9700
C14—Cl2	1.738 (4)	C31—H31B	0.9700
C15—C16	1.386 (5)	C32—C33	1.380 (13)
C15—H15	0.9300	C32—H32A	0.9700
C16—H16	0.9300	C32—H32B	0.9700
C17—O1	1.226 (3)	C33—O3	1.328 (9)
C17—N3	1.350 (4)	C33—H33A	0.9700
C17—C18	1.493 (4)	C33—H33B	0.9700
C18—H18A	0.9600	C4—H4A	0.9300
C18—H18B	0.9600	N2—N3	1.389 (3)
C18—H18C	0.9600	N4—N5	1.384 (3)
			
C4—C1—C2	117.2 (2)	N5—C20—H20	109.5
C4—C1—C8	118.8 (2)	C24—C20—H20	109.5
C2—C1—C8	123.9 (3)	C21—C20—H20	109.5
N1—C2—C1	121.3 (3)	C19—C21—C20	103.3 (2)
N1—C2—C3	113.9 (3)	C19—C21—H21A	111.1
C1—C2—C3	124.7 (3)	C20—C21—H21A	111.1
C2—C3—H3A	109.5	C19—C21—H21B	111.1
C2—C3—H3B	109.5	C20—C21—H21B	111.1
H3A—C3—H3B	109.5	H21A—C21—H21B	109.1
C2—C3—H3C	109.5	O2—C22—N5	119.4 (3)
H3A—C3—H3C	109.5	O2—C22—C23	124.3 (3)
H3B—C3—H3C	109.5	N5—C22—C23	116.2 (3)
C5—N1—C2	121.2 (2)	C22—C23—H23A	109.5
N1—C5—C7	121.1 (2)	C22—C23—H23B	109.5
N1—C5—C6	114.0 (3)	H23A—C23—H23B	109.5
C7—C5—C6	124.9 (3)	C22—C23—H23C	109.5
C5—C6—H6A	109.5	H23A—C23—H23C	109.5
C5—C6—H6B	109.5	H23B—C23—H23C	109.5
H6A—C6—H6B	109.5	C25—C24—C29	118.3 (3)
C5—C6—H6C	109.5	C25—C24—C20	121.5 (3)
H6A—C6—H6C	109.5	C29—C24—C20	120.2 (3)
H6B—C6—H6C	109.5	C24—C25—C26	121.3 (3)
C4—C7—C5	117.0 (3)	C24—C25—H25	119.4
C4—C7—C19	118.9 (3)	C26—C25—H25	119.4
C5—C7—C19	124.1 (2)	C27—C26—C25	118.8 (4)
N2—C8—C1	122.9 (2)	C27—C26—H26	120.6
N2—C8—C9	113.8 (2)	C25—C26—H26	120.6
C1—C8—C9	123.3 (3)	C26—C27—C28	121.7 (4)
C8—C9—C10	103.6 (2)	C26—C27—Cl1	119.5 (4)
C8—C9—H9A	111.0	C28—C27—Cl1	118.8 (4)
C10—C9—H9A	111.0	C27—C28—C29	119.2 (3)
C8—C9—H9B	111.0	C27—C28—H28	120.4
C10—C9—H9B	111.0	C29—C28—H28	120.4
H9A—C9—H9B	109.0	C24—C29—C28	120.7 (4)
N3—C10—C11	113.0 (2)	C24—C29—H29	119.7
N3—C10—C9	101.0 (2)	C28—C29—H29	119.7
C11—C10—C9	114.8 (3)	C31—C30—O3	104.8 (8)
N3—C10—H10	109.2	C31—C30—H30A	110.8
C11—C10—H10	109.2	O3—C30—H30A	110.8
C9—C10—H10	109.2	C31—C30—H30B	110.8
C16—C11—C12	117.2 (3)	O3—C30—H30B	110.8
C16—C11—C10	121.1 (3)	H30A—C30—H30B	108.9
C12—C11—C10	121.7 (3)	C30—C31—C32	102.7 (8)
C11—C12—C13	121.6 (3)	C30—C31—H31A	111.2
C11—C12—H12	119.2	C32—C31—H31A	111.2
C13—C12—H12	119.2	C30—C31—H31B	111.2
C14—C13—C12	119.5 (3)	C32—C31—H31B	111.2
C14—C13—H13	120.2	H31A—C31—H31B	109.1
C12—C13—H13	120.2	C33—C32—C31	103.5 (8)
C13—C14—C15	120.6 (3)	C33—C32—H32A	111.1
C13—C14—Cl2	119.8 (3)	C31—C32—H32A	111.1
C15—C14—Cl2	119.6 (3)	C33—C32—H32B	111.1
C14—C15—C16	118.9 (3)	C31—C32—H32B	111.1
C14—C15—H15	120.5	H32A—C32—H32B	109.0
C16—C15—H15	120.5	O3—C33—C32	93.9 (7)
C11—C16—C15	122.2 (3)	O3—C33—H33A	113.0
C11—C16—H16	118.9	C32—C33—H33A	113.0
C15—C16—H16	118.9	O3—C33—H33B	113.0
O1—C17—N3	119.2 (3)	C32—C33—H33B	113.0
O1—C17—C18	123.2 (3)	H33A—C33—H33B	110.4
N3—C17—C18	117.6 (3)	C1—C4—C7	122.1 (3)
C17—C18—H18A	109.5	C1—C4—H4A	119.0
C17—C18—H18B	109.5	C7—C4—H4A	119.0
H18A—C18—H18B	109.5	C8—N2—N3	108.5 (2)
C17—C18—H18C	109.5	C17—N3—N2	121.8 (2)
H18A—C18—H18C	109.5	C17—N3—C10	124.5 (3)
H18B—C18—H18C	109.5	N2—N3—C10	113.0 (2)
N4—C19—C7	122.2 (3)	C19—N4—N5	109.4 (2)
N4—C19—C21	113.0 (3)	C22—N5—N4	120.6 (3)
C7—C19—C21	124.8 (2)	C22—N5—C20	124.7 (3)
N5—C20—C24	111.6 (3)	N4—N5—C20	113.3 (2)
N5—C20—C21	100.6 (2)	C33—O3—C30	100.0 (7)
C24—C20—C21	115.8 (3)		
			
C4—C1—C2—N1	0.7 (4)	C21—C20—C24—C29	−127.4 (3)
C8—C1—C2—N1	−176.9 (3)	C29—C24—C25—C26	−1.4 (5)
C4—C1—C2—C3	179.5 (3)	C20—C24—C25—C26	177.1 (3)
C8—C1—C2—C3	1.9 (5)	C24—C25—C26—C27	0.1 (6)
C1—C2—N1—C5	−0.6 (4)	C25—C26—C27—C28	1.6 (6)
C3—C2—N1—C5	−179.6 (3)	C25—C26—C27—Cl1	−177.6 (3)
C2—N1—C5—C7	−0.4 (4)	C26—C27—C28—C29	−1.9 (6)
C2—N1—C5—C6	179.4 (3)	Cl1—C27—C28—C29	177.3 (3)
N1—C5—C7—C4	1.2 (4)	C25—C24—C29—C28	1.1 (5)
C6—C5—C7—C4	−178.5 (3)	C20—C24—C29—C28	−177.5 (3)
N1—C5—C7—C19	−178.3 (3)	C27—C28—C29—C24	0.5 (6)
C6—C5—C7—C19	2.0 (5)	O3—C30—C31—C32	−18.4 (14)
C4—C1—C8—N2	167.5 (3)	C30—C31—C32—C33	−17.0 (16)
C2—C1—C8—N2	−14.9 (4)	C31—C32—C33—O3	45.8 (12)
C4—C1—C8—C9	−13.4 (4)	C2—C1—C4—C7	0.2 (4)
C2—C1—C8—C9	164.2 (3)	C8—C1—C4—C7	178.0 (2)
N2—C8—C9—C10	−2.2 (4)	C5—C7—C4—C1	−1.1 (4)
C1—C8—C9—C10	178.7 (3)	C19—C7—C4—C1	178.4 (2)
C8—C9—C10—N3	2.6 (3)	C1—C8—N2—N3	179.7 (2)
C8—C9—C10—C11	−119.2 (3)	C9—C8—N2—N3	0.6 (3)
N3—C10—C11—C16	−49.3 (4)	O1—C17—N3—N2	175.8 (3)
C9—C10—C11—C16	65.7 (4)	C18—C17—N3—N2	−3.2 (4)
N3—C10—C11—C12	130.7 (3)	O1—C17—N3—C10	6.3 (5)
C9—C10—C11—C12	−114.2 (3)	C18—C17—N3—C10	−172.7 (3)
C16—C11—C12—C13	0.4 (5)	C8—N2—N3—C17	−169.2 (3)
C10—C11—C12—C13	−179.7 (3)	C8—N2—N3—C10	1.4 (3)
C11—C12—C13—C14	0.1 (6)	C11—C10—N3—C17	−69.2 (4)
C12—C13—C14—C15	−0.8 (6)	C9—C10—N3—C17	167.7 (3)
C12—C13—C14—Cl2	178.1 (3)	C11—C10—N3—N2	120.5 (3)
C13—C14—C15—C16	1.0 (5)	C9—C10—N3—N2	−2.6 (3)
Cl2—C14—C15—C16	−177.8 (3)	C7—C19—N4—N5	178.8 (2)
C12—C11—C16—C15	−0.1 (5)	C21—C19—N4—N5	−1.0 (4)
C10—C11—C16—C15	179.9 (3)	O2—C22—N5—N4	168.8 (3)
C14—C15—C16—C11	−0.6 (5)	C23—C22—N5—N4	−14.0 (5)
C4—C7—C19—N4	177.9 (3)	O2—C22—N5—C20	3.5 (6)
C5—C7—C19—N4	−2.6 (4)	C23—C22—N5—C20	−179.3 (3)
C4—C7—C19—C21	−2.4 (4)	C19—N4—N5—C22	−170.4 (3)
C5—C7—C19—C21	177.1 (3)	C19—N4—N5—C20	−3.6 (3)
N4—C19—C21—C20	4.7 (4)	C24—C20—N5—C22	−64.2 (4)
C7—C19—C21—C20	−175.0 (3)	C21—C20—N5—C22	172.4 (3)
N5—C20—C21—C19	−6.0 (3)	C24—C20—N5—N4	129.5 (3)
C24—C20—C21—C19	−126.5 (3)	C21—C20—N5—N4	6.1 (3)
N5—C20—C24—C25	−60.2 (4)	C32—C33—O3—C30	−56.7 (11)
C21—C20—C24—C25	54.1 (4)	C31—C30—O3—C33	50.4 (13)
N5—C20—C24—C29	118.3 (3)		

### Hydrogen-bond geometry (Å, °)

**Table:**

*D*—H···*A*	*D*—H	H···*A*	*D*···*A*	*D*—H···*A*
C9—H9B···O3	0.97	2.49	3.423 (7)	163
C21—H21B···O1^i^	0.97	2.57	3.211 (4)	123
C9—H9A···O1^i^	0.97	2.35	3.234 (4)	151
C23—H23A···O2^ii^	0.96	2.45	3.385 (4)	166

Symmetry codes: (i) −*x*+1, *y*+1/2, −*z*+1/2; (ii) −*x*+2, −*y*+2, −*z*.
